# Complement component C1q initiates extrinsic coagulation via the receptor for the globular head of C1q in adventitial fibroblasts and vascular smooth muscle cells

**DOI:** 10.1002/iid3.769

**Published:** 2023-01-23

**Authors:** Christopher T. Freda, Wei Yin, Berhane Ghebrehiwet, David A. Rubenstein

**Affiliations:** ^1^ Department of Biomedical Engineering Stony Brook University Stony Brook New York USA; ^2^ Department of Medicine Stony Brook University Stony Brook New York USA

**Keywords:** complement, gC1qR, inflammation, thrombosis, tissue factor

## Abstract

**Background:**

Vascular diseases are highly associated with inflammation and thrombosis. Elucidating links between these two processes may provide a clearer understanding of these diseases, allowing for the design of more effective treatments. The activation of complement component 1 (C1) is a crucial contributor to innate immunity and is associated with significant concentrations of circulating C1q. Many pathological pathways initiate when C1q interacts with gC1qR. This interaction plays a major role in inflammation observed during atherosclerosis and the initiation of intrinsic coagulation. However, the effects of C1 and the role of C1q/gC1qR on extrinsic coagulation, which is the more physiologically relevant coagulation arm, has not been studied. We hypothesized that C1q binding to gC1qR enhances the expression of tissue factor (TF) in adventitial fibroblasts and vascular smooth muscle cells, the primary TF bearing cells in the body.

**Methods:**

Using an enzyme‐linked immunosorbent assay approach, TF expression and the role of gC1qR was observed. Cells were conditioned for 1 h with C1q or a gC1qR blocker and C1q, to assess the role of gC1qR. Additionally, cell growth characteristics were monitored to assess changes in viability and metabolic activity.

**Results:**

Our results indicate that the expression of TF increased significantly after incubation with C1q as compared with unconditioned cells. Cells conditioned with gC1qR blockers and C1q exhibited no change in TF expression when compared with cells conditioned with the blocking antibodies alone. Our results show no significant differences in metabolic activity or cell viability under these conditions.

**Conclusions:**

This indicates that gC1qR association with C1q induces TF expression and may initiate extrinsic coagulation. Overall, this data illustrates a role for C1q in the activation of extrinsic coagulation and that gC1qR activity may link inflammation and thrombosis.

## Graphical abstract

Vascular diseases are associated with altered inflammation and thrombosis. We have identified a link between complement activity and activation of extrinsic coagulation.

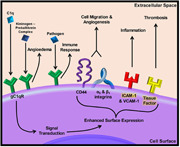

## 1 INTRODUCTION

Cardiovascular diseases encompass a wide range of complications associated with the heart and blood vessels and have persisted as the number one cause of death in the world over the last 2 decades.^1^, ^2^ Narrowing of the blood vessels due to plaque buildup within the vessel walls, or atherosclerosis, is one of the most common complications associated with vascular diseases. This can lead to severe reductions in blood flow to downstream tissue or a complete blockage of blood flow from the heart.^3^ Conventionally, atherosclerosis has been considered vascular in nature; however, it has recently become apparent that atherosclerosis can be characterized by changes in both the thrombotic and inflammatory responses and that these changes can have wide‐ranging systemic effects.^4^, ^5^

The result of physiological or pathological coagulation can cause blood vessels to become obstructed through the formation of a thrombus. The intrinsic and extrinsic coagulation cascades can each result in thrombus formation; however, it is the extrinsic cascade that has more physiological relevance. Extrinsic coagulation initiates upon vessel damage, exposing tissue‐factor‐bearing cells, such as vascular smooth muscle cells (SMCs) and adventitial fibroblasts (AFs), to blood constitutents.^6^, ^7^ Circulating Factor VII binds to exposed tissue factor, initiating coagulation through this complex's ability to enzymatically activate Factor X.^8^

Both innate and adaptive immune responses play an essential role in the development of cardiovascular diseases and its complications.^4^, ^9^, ^10^ Physiological inflammation is a common defense mechanism in response to infection or cell injury upon stimulation by inflammatory cytokines or the secretion of chemical factors by the injured cells. The inflammatory response can also be influenced by the complement system, which is a cascade of enzymatic reactions that results in the formation of the membrane attack complex and the lysis of invading pathogens. Complement component 1q (C1q) is one of the initiating components of the complement system. In addition to binding to antigen‐antibody complexes,^11^, ^12^ C1q has an affinity for gC1qR, a ubiquitous cell surface receptor that is found on cell types associated with cardiovascular disease development. Typically, the binding of C1q to gC1qR induces immune responses, including mediation of infection,^13^ phagocytosis and uptake of apoptotic cells,^13^, ^14^ monocyte differentiation,^15^ and inflammation.^16^ However, studies have also found this binding to be associated with autoimmune diseases, infection, intrinsic coagulation, endothelial cell activation, COVID‐19 and carcinogenesis.^17^, ^18^, ^19^, ^20^, ^21^ It is also well‐cited that C1q is present at sites of atherosclerosis and inflammatory and vascular lesions,^22^, ^23^, ^24^ suggesting a strong role for complement in vascular diseases, however, the significance of its presence has yet to be elucidated. More specifically, complement has been shown to stimulate platelet and endothelial cell activities,^25^, ^26^, ^27^ however, the extent of these interactions has not been fully characterized. While some links have been established between complement and coagulation through C1q‐gC1qR association, it is unclear what the initiating steps are, their relevance in disease progression, and how these steps can be targeted to mitigate disease responses. Recent findings also show that complement component 1 (C1) is activated during early pathological cardiovascular events, leading to increased levels of circulating C1q.^28^ C1q has also been found in the sub‐endothelial space under these conditions and has been shown to influence vascular smooth muscle cell functions related to proliferation.^29^ Thus, it is critical to evaluate the effect of C1q on vascular inflammatory processes.

We chose to investigate the role of the C1q‐gC1qR axis as a link between inflammation and coagulation to promote extrinsic coagulation activation. Characterization of this new link would provide new avenues for vascular disease research and identify new targets for therapeutic intervention strategies.

## 2 MATERIALS AND METHODS

### 2.1 Cells

Human AFs and human coronary artery SMCs were purchased from ScienCell Research Laboratories and Cell Applications Inc, respectively. AFs were kept in fibroblast medium‐2 supplemented with 5% fetal bovine serum, antibiotics (penicillin/streptomycin), and growth supplement (as suggested by and purchased from ScienCell Research Laboratories) at 37°C and 5% CO_2_. SMCs were kept in smooth muscle cell medium supplemented with 2% fetal bovine serum, antibiotics (penicillin/streptomycin), and growth supplement (as suggested by and purchased from Cell Applications) at 37°C and 5% CO_2_. Both cell types were cultured on tissue culture plastic flasks and well‐plates. At confluence, cells were passaged with trypsin digestion for approximately 2 min at room temperature (note that all reagents were purchased from Millipore Sigma, unless noted otherwise). For experiments, cells were incubated with purified human C1q (Quidel Corporation), lipopolysaccharides from *Escherichia coli* (LPS), human platelet‐poor plasma (PPP, 1:10 in HEPES‐buffered Tyrode's solution, pH 7.4), or a combination of anti‐gC1qR (60.11, C1q binding site, from B. Ghebrehiwet) and C1q, LPS, or PPP. All experiments also included an internal negative control consisting of cells exposed only to media for the entire duration. For statistical purposes, cell seeding density was maintained at ~40,000 cells/cm^2^ for all experiments. Note that all appropriate ethical guidelines were followed during this study.

### 2.2 Cell viability, density, and metabolic activity

To determine if the incubation of cells with the particular experimental conditions induced changes in cell culture parameters, we used a standard live/dead cell cytotoxicity assay and the MTT assay to quantify cell viability, density, and metabolic activity after the exposure conditions. The live/dead cell cytotoxicity assay consisted of 2 μM calcein and 4 μM ethidium (Thermo Fisher Scientific). After the cells were incubated in the exposure conditions, they were washed with warmed PBS (pH 7.4, 37°C) and immediately incubated with ~50–100 μL of the calcein/ethidium mixture for 3 min. After this incubation, cells were immediately imaged on an inverted microscope at three randomized locations per independent tissue culture well (Nikon, TE‐2000U). The data from each well was then averaged for a single data point. Cell viability was calculated by dividing the number of live cells in the imaging area by the total number of cells per imaging area.^30^ Cell density is the total number of live cells per imaging area, calibrated for each of our microscope objectives. The cell density was then normalized by the cell seeding density to provide a measure of proliferation over the time interval.^30^

To quantify the metabolic activity of each cell type after exposure conditions, a 3‐[4,5‐dimethylthiazol‐2‐yl]−2,5‐diphenyl tetrazolium bromide (MTT) assay was used. This assay quantifies the activity of mitochondrial dehydrogenase (all MTT reagents from Millipore Sigma). After the experimental time course, cells were washed with warmed PBS (pH 7.4, 37°C) and then immediately incubated with MTT reagent reconstituted in appropriate basal media for approximately 2 h. Formazan crystals were dissolved in 10% Triton‐X and 0.1 M HCl in anhydrous isopropanol. The ensuing solution was mixed on a platform rocker for approximately 15 min. Duplicate 100 μL samples were collected from each independent condition and transferred to a 96‐well plate for absorbance measurements and to ensure data accuracy. The absorbance resulting from the dissolving procedures was quantified at 570 nm using a microplate reader (SpectraMax i3, Molecular Devices; note that this microplate reader was used for all absorbance data collection). All data was normalized to the metabolic activity of paired wells incubated without exposure conditions.^30^

### 2.3 Tissue factor and ICAM‐1 expression

Tissue factor (TF) and ICAM‐1 expression on both cell types was quantified after the exposure conditions using a solid‐phase ELISA approach. Cells were washed with warmed PBS (pH 7.4, 37°C), fixed with 0.5% glutaraldehyde (15 min, pH 7.4, 37°C), then neutralized with 100 mM glycine –0.1% BSA (30 min, pH 7.5). TF and ICAM‐1 expression were assessed with an anti‐CD142 monoclonal antibody and an anti‐ICAM‐1 monoclonal antibody (both purchased from Invitrogen), respectively. Cells were incubated with the respective primary antibody for 1 h at a final concentration of 1 μg/mL. To detect primary antibody binding, cells were then washed with PBS and incubated with an appropriate alkaline phosphatase conjugated secondary antibody for 1 h at a concentration of 1 μg/mL. Color development was achieved by addition of pNPP, and absorbance was read spectrophotometrically at 405 nm using a microplate reader. Note that all details have been reported by us previously and that all appropriate negative and positive controls were included within each independent experiment.^30^, ^31^

### 2.4 Tissue factor expression while blocking gC1qR

To investigate the potential mechanism for C1q‐induced TF expression, we used a similar ELISA approach as described above. Briefly, TF expression was quantified after 1 h of exposure conditions. Before incubating the cells with the experimental conditions (as described above), cells were first treated with a monoclonal antibody for gC1qR (60.11 region) for 1 h. Cells were then washed with warmed PBS (37°C, pH 7.4), fixed with 0.5% glutaraldehyde (15 min, pH 7.4, 37°C), then neutralized with 100 mM glycine –0.1% BSA (30 min, pH 7.5). Cells were then incubated with an anti‐CD142 (TF) antibody for 1 h at a final concentration of 1 μg/mL. To detect antibody binding, cells were washed with PBS then incubated with an appropriate alkaline phosphatase secondary antibody for 1 h at a concentration of 1 μg/mL. Color development was achieved by addition of pNPP, and absorbance was observed spectrophotometrically at 405 nm using a microplate reader.

### 2.5 Secreted tissue factor

The presence of secreted tissue factor, in response to C1q, was observed using a tissue factor capture ELISA kit (Millipore Sigma). A TF standard curve was generated to convert absorbance readings into TF concentration. After the cells were incubated with the experimental conditions for 1 h, 100 μL of the conditioned media was transferred to a plate containing the TF capture antibody and plates were placed on a platform rocker for 2.5 h with gentle rocking at room temperature (25°C). The plate was then washed using the supplied wash buffer. The detection antibody was then added for 1 h at room temperature with gentle rocking. The plate was washed again, then a streptavidin‐HRP conjugated antibody was added to each well for 45 min at room temperature with gentle rocking. A TMB One‐Step Reagent was then added to each well for 30 min, followed by the provided stop solution. Absorbance was observed spectrophotometrically at 450 nm immediately using a microplate reader. The absorbance readings of the known standard values of tissue factor were used to convert the absorbance readings of our samples to the amount of released tissue factor that was present.

### 2.6 Statistics

All viability, density, and metabolic activity data was normalized as described above. All ELISA data from each independent experiment was normalized to the paired control samples (e.g., cells incubated for the same duration in pure media), with background subtraction, as appropriate. Note that since there were no statistical differences in the culture conditions after the experimental conditions, we did not normalize ELISA data by cell growth characteristics. All experiments were conducted with multiple dependent technical replicates (*n* = 2–3). The dependent data was first averaged to obtain a single independent data point for the particular experimental condition. Normalized data from at least three independent experiments are shown and used for statistical analysis (all sample size numbers are reported in the Figure legends). Statistical analysis was carried out in SAS (v 9.4, SAS Institute) using a one‐way analysis of variance (ANOVA) procedure (factor is the incubation condition) and the Duncan post‐hoc test. Note that all exposures were compared to a negative control (termed “Basal Media”), which was cells incubated for the appropriate duration in standard cell culture media.

## 3 RESULTS

### 3.1 Cell viability, density, and metabolic activity

**Figure 1:**
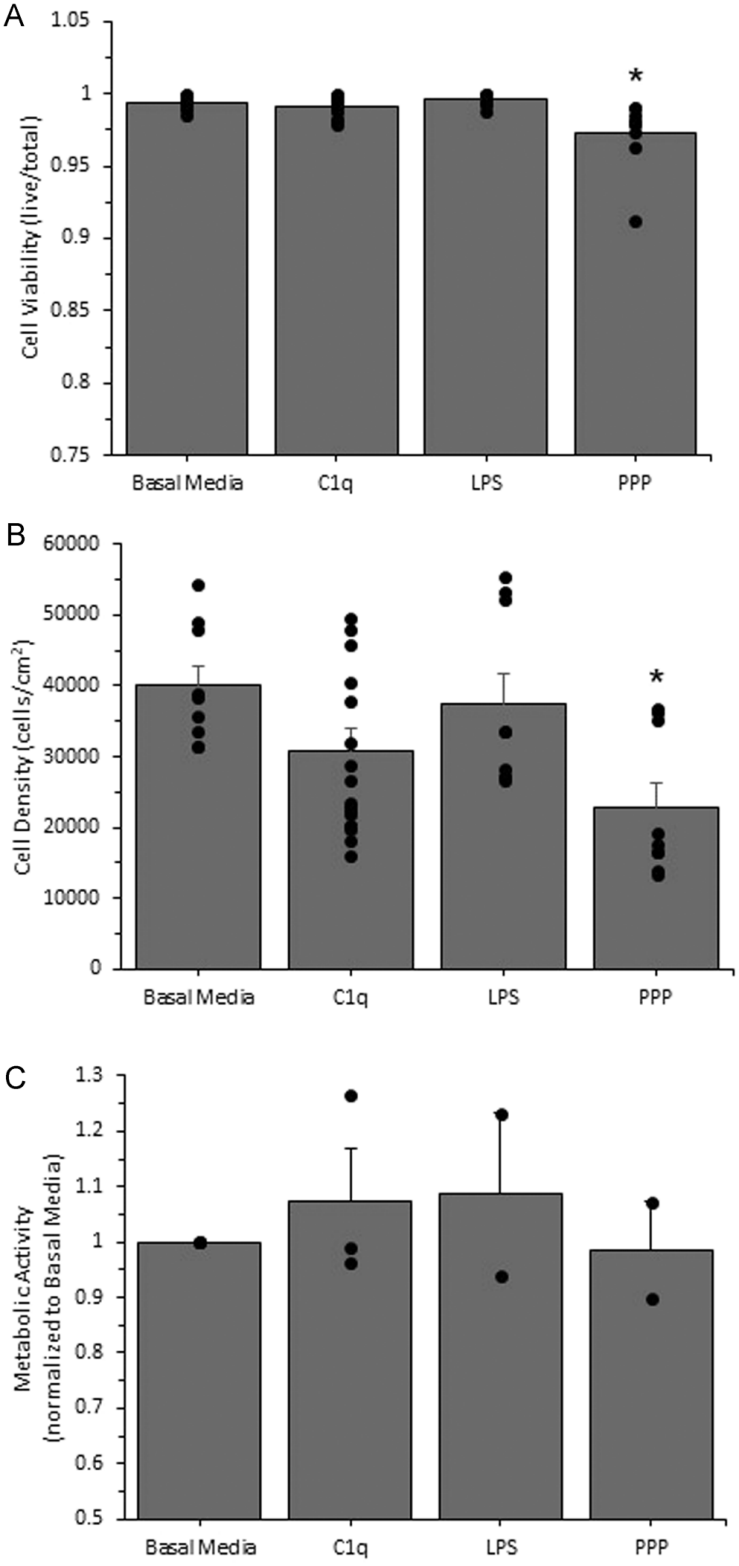
Human aortic adventitial fibroblast cell viability (A), density (B) and metabolic activity (C) after a short duration exposure to human C1q, LPS, or platelet poor plasma (PPP). Viability and density were measured with a standard live/dead cell cytotoxicity assay and metabolic activity was measured with the MTT assay, using absorbance and immunofluorescence microscopy as described in the materials and methods section. Cells were incubated with the various conditions and then assessed for surface expression. All data is reported as the mean + standard error of the mean from 2 to 18 independent experiments (each independent experiment included at least two technical repeats; cell viability and cell density *n* = 9–18; metabolic activity *n* = 2–4). Independent data points are marked with closed circles for each condition. *Significantly different than negative control (analysis of variance, Duncan method, *p* < .05).

**Figure 2:**
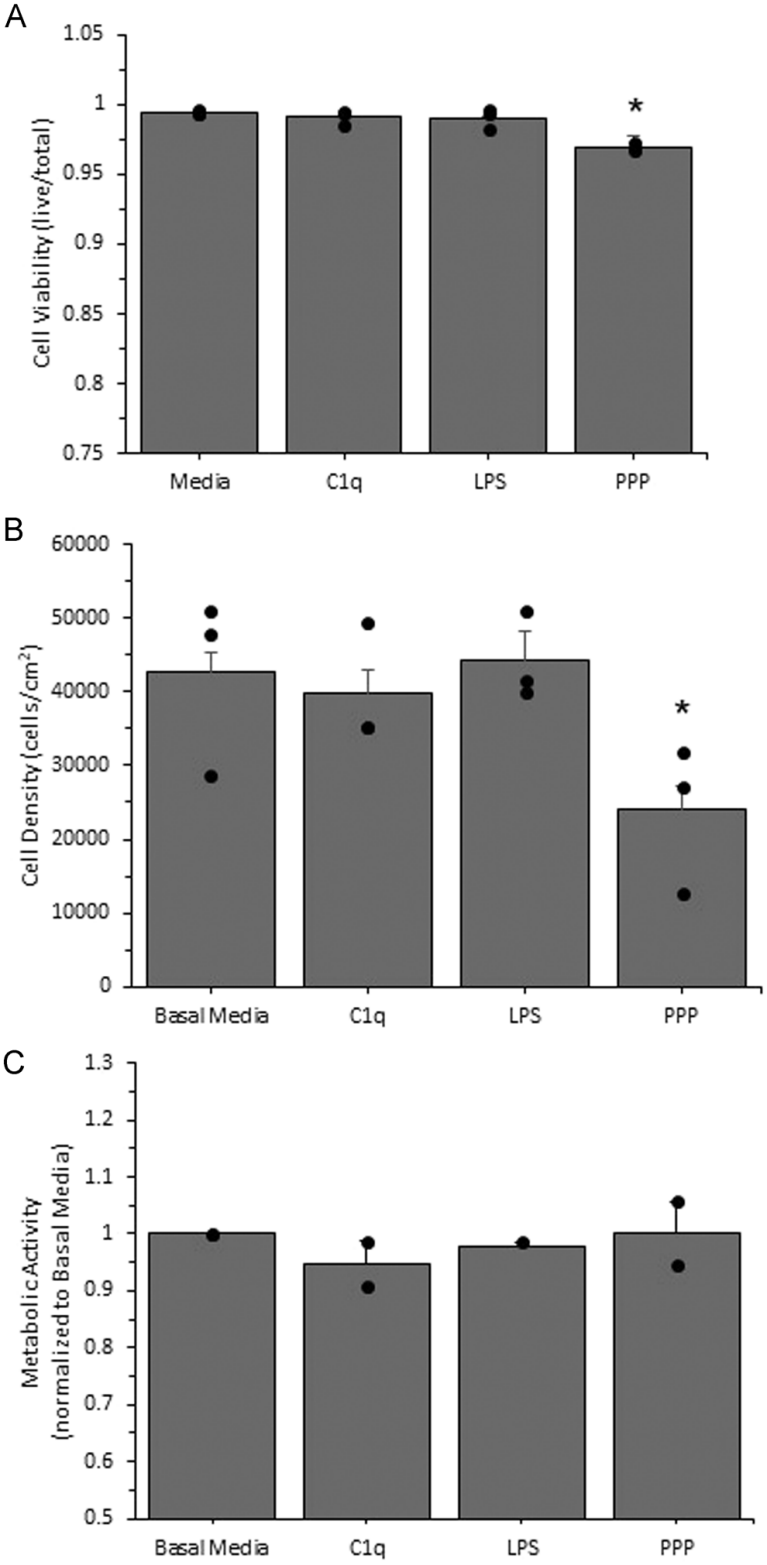
Human coronary artery smooth muscle cell viability (A), density (B) and metabolic activity (C) after a short duration exposure to human C1q, LPS, or platelet poor plasma (PPP). Viability and density were measured with a standard live/dead cell cytotoxicity assay and metabolic activity was measured with the MTT assay, using absorbance and immunofluorescence microscopy as described in the materials and methods section. Cells were incubated with the various conditions and then assessed for surface expression. All data is reported as the mean + standard error of the mean from 2 to 3 independent experiments (each independent experiment included at least two technical repeats; cell viability and cell density *n* = 3; metabolic activity *n* = 2). Independent data points are marked with closed circles for each condition. *Significantly different than negative control (analysis of variance, Duncan method, *p* < .05).

To determine the effects of C1q on AF (Figure 1) or SMC (Figure 2) culture conditions, we quantified cell viability, density, and metabolic activity. Cell viability is a measure of cell death in response to our exposure conditions. Following 24‐hour exposure with C1q, we observed little to no changes in cell viability (Figures 1A [AF] and 2A [SMC]). Cell density is a measure of whether or not the exposure conditions have an effect on cell proliferation. After a 24‐h incubation with C1q, we observed no changes in cell density as compared to our negative control (Figures 1B [AF] and 2B [SMC]). There was also no statistically significant change in metabolic activity following exposure to C1q as compared to our negative control (Figures 1C [AF] and 2C [SMC]). These data indicate that C1q does not directly interfere with cell viability, cell growth, and metabolic activity over a 24‐h period. Note that the exposure to 1:10 PPP significantly reduce cell viability and cell density for both cell types. Since there was no change in culture parameters following C1q incubation, no further normalization of ELISA data was conducted.

### 3.2 Tissue factor and ICAM‐1 expression

**Figure 3:**
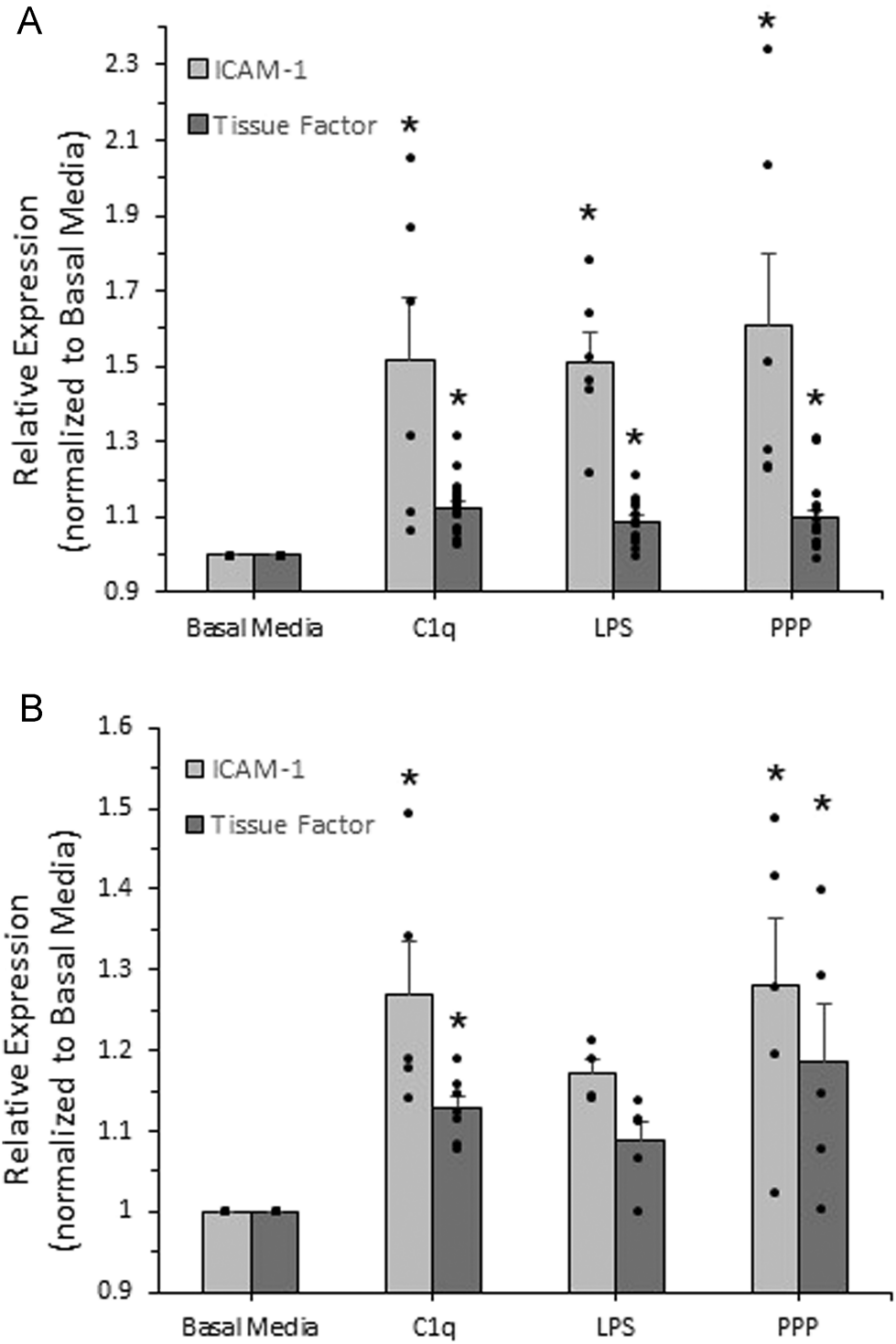
Human aortic adventitial fibroblast (A) and human coronary artery smooth muscle cell (B) gC1qR (A), ICAM‐1 (A), and tissue factor (A,B) surface expression after a short duration exposure to C1q, LPS, or platelet poor plasma (PPP). Using an enzyme‐linked immunosorbent assay approach, the surface expression of the ICAM‐1 and tissue factor were assessed, as described in the methods section. Cells were incubated with the various conditions and then assessed for surface expression. All data is reported as the mean + standard error of the mean for a minimum of four independent experiments (each independent experiment included at least two technical repeats; Figure 3A ICAM‐1, *n* = 6–7; Figure 3A tissue factor, *n* = 16–24; Figure 3B ICAM‐1, *n* = 4–6; Figure 3B Tissue Factor *n* = 5–7). Independent data points are marked with closed circles for each condition. *Significantly different than negative control (analysis of variance, Duncan method, *p* < .05).

Quantification of both coagulation and inflammatory response markers was performed. After a 1‐h exposure to C1q, we observed an elevated expression for both markers as compared with cells that were exposed to basal media. In particular, the expression of TF and ICAM‐1 was significantly enhanced in AFs after exposure to C1q (Figure 3A, ANOVA, *p* < .05). Additionally, when SMCs were conditioned with C1q for 1 h, there was a significant increase in the expression of both tissue factor and ICAM‐1. (Figure 3B, ANOVA, *p* < .05). It is important to note that for AFs, the increase in tissue factor and ICAM‐1 expression, after exposure to C1q, mimicked the increases observed after exposure to our positive controls. Similarly, for SMCs, the exposure to C1q significantly increased ICAM‐1 and tissue factor expression to similar levels as PPP exposure.

### 3.3 Tissue factor expression while blocking gC1qR

**Figure 4:**
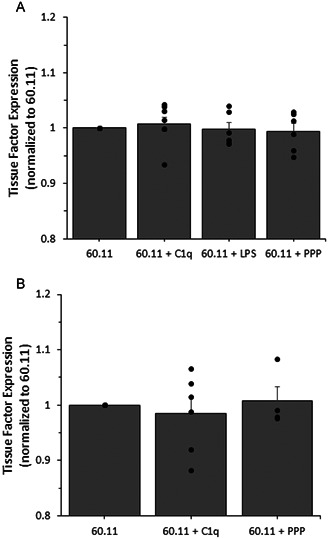
Human aortic adventitial fibroblast (A) and human coronary artery smooth muscle cell (B) tissue factor expression after a short duration exposure to gC1qR blocking antibodies (60.11) followed by a short duration exposure to either C1q, LPS, or platelet poor plasma (PPP). Using an enzyme‐linked immunosorbent assay approach, the surface expression of the ICAM‐1 and tissue factor were assessed, as described in the methods section. Cells were incubated with the various conditions and then assessed for surface expression. No significant changes were observed, indicating successful blocking of gC1qR and indication that C1q binding to gC1qR induces changes in tissue factor expression. All data is reported as the mean + standard error of the mean for a minimum of six independent experiments (each independent experiment included at least two technical repeats). Independent data points are marked with closed circles for each condition.

After confirming the increase in tissue factor expression in both adventitial fibroblasts and coronary artery smooth muscle cells in response to C1q exposure, we observed TF expression while blocking C1q association with gC1qR. A monoclonal antibody (60.11) that specifically and selectively targets the C1q binding domain of gC1qR was used. After a 1‐h incubation with the blocking antibody, followed by a 1‐h incubation with C1q, there was no statistically significant change in the expression of tissue factor when compared to cells that were treated with the blocking antibody but not C1q (Figure 4A [AF] and 4B [SMC]). For SMCs, we did not quantify 60.11 + LPS, since LPS itself could not elicit tissue factor expression. Further, since ICAM‐1 was used as a confirmatory inflammatory marker, we did not observe its expression under these conditions. It is also important to note, that under these conditions, there was no increase in tissue factor observed for any of our experimental conditions.

### 3.4 Secreted tissue factor

**Figure 5:**
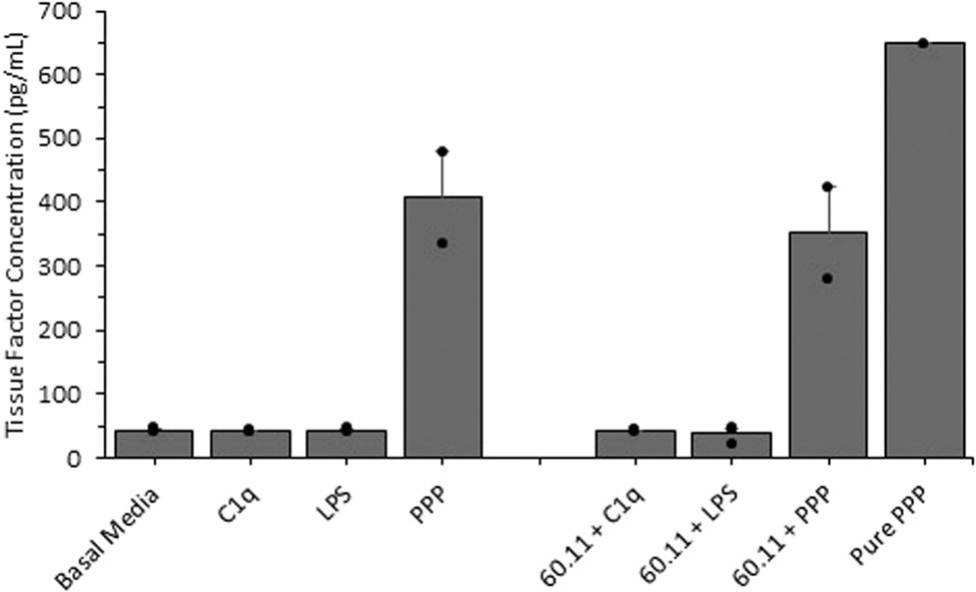
Secretion of tissue factor from human aortic adventitial fibroblast in the presence or absence of gC1qR blocking antibodies (60.11). C1q, LPS, or platelet poor plasma (PPP) was used to antagonize tissue factor secretion. Using an enzyme‐linked immunosorbent assay approach, the secretion of tissue factor was assessed, as described in the methods section. Cells were incubated with the various conditions and then assessed for tissue factor secretion. No significant changes were observed. Data is reported as the mean + standard error of the mean from 2 to 3 independent experiments. Independent data points are marked with closed circles for each condition.

In order to quantify if cells released soluble tissue factor in response to C1q, in lieu of surface‐bound tissue factor, a capture ELISA was used to determine TF concentration within media after the experimental conditions. After exposing cells to C1q (as above), secreted tissue factor was found to be at a concentration less than 50 pg/ml (Figure 5); nearly identical to our negative control (cells with media only, “Basal Media”), indicating that there were no changes in secreted tissue factor when the cells were exposed to C1q. As a confirmation, we observed the role of blocking gC1qR on the secretion of tissue factor. There were no differences between tissue factor secretion in the presence/absence of gC1qR blocking antibodies. As a confirmation we also observed the concentration of tissue factor in our PPP samples (labeled as “Pure PPP,” Figure 5) and observed a concentration around 1300–1400 pg/ml.

## 4 DISCUSSION

### 4.1 Cell viability, density, and metabolic activity

To determine whether or not C1q alters AF cell survival and growth, we quantified cell viability and density (Figure 1A,B, respectively) after 24 h of exposure. Additionally, cell viability and density for SMCs was also quantified (Figure 2A,B, respectively) after 1 h of exposure. Our data suggests C1q has no direct effect on cell survival nor growth. Although C1q has been shown to be associated with a wide variety of positive and negative cellular activities in liver^32^ and prostate cancer cells,^33^ embryonic kidney cells,^32^ murine fibroblasts,^32^ and human vascular smooth muscle cells,^34^ and has also been shown to alter human fibroblast cell growth after stimulation with platelet derived growth factor,^35^ a direct association between C1q and cell viability has not been cited. Our findings suggest that C1q exposure does not influence cell viability or density in both AFs and SMCs. Although C1q plays an important role in apoptosis by recognizing and binding to apoptotic cells and initiating the complement cascade of the immune system,^36^ which ultimately results in cell death, this has not been observed in cell culture where subsequent complement proteins are not in abundance. C1q has not been shown to promote cell death or induce apoptosis in healthy cultured cells. Although C1q has been shown to regulate mitochondrial metabolism in memory precursor effector cells^37^ and stimulate adenylyl cyclase activity of human fibroblasts,^38^ a direct association between C1q and the metabolic activity of AFs/SMCs has not been reported. We examined the metabolic activity of AFs and SMCs to determine whether or not C1q has an effect on the metabolism of these cells. Our data suggests that exposure to C1q does not alter metabolic activity in AFs nor SMCs (Figures 1C and 2C, respectively). These findings allowed us to conclude that there were no changes to the culture conditions, due to the exposure to C1q and agree with the majority of the previous reports relating these cell functions.

### 4.2 Tissue factor and ICAM‐1 expression

The expression of tissue factor and ICAM‐1 was assessed to determine whether the exposure to C1q alters the expression of these markers. Our data suggests that both of these markers are sensitive to the presence of C1q in AFs and SMCs (Figure 3A,B, respectively). Enhanced tissue factor expression can lead to the increased association with factor VII (FVII), activation of FVII and the resulting formation of the enzymatically active TF:FVIIa complex. In the presence of Factor X, an active TF:FVIIa complex, would allow for the progression of coagulation through the initiation of the extrinsic coagulation arm.^7^ Complement activation has been shown to enhance the expression of tissue factor on intravascular cells.^39^ Our data extends this knowledge to extravascular cells, specifically via the presence of circulating C1q. There have been many other findings that support a link between the complement system and the coagulation cascade at various points along both pathways.^25^, ^40^, ^41^ For example, one group has found that in the absence of complement component C3, which is normally required to activate C5, thrombin alone was able to generate activated C5a.^42^ It has also been shown that both C3 and C5 can be activated by thrombin, factor XIa, Xa, and IXa.^43^ Also, Hageman factor (FXII) is able to activate the C1 complex, initiating the classical pathway of the complement system.^44^ Furthermore, complement component C1q has been associated with platelet activation, indicating a role in coagulation.^45^, ^46^ Overall, our data suggests that when AFs and SMCs are exposed to C1q, this can stimulate the initiation of extrinsic coagulation via the increased expression of tissue factor, providing a new link between inflammation and coagulation.

Intercellular adhesion molecule‐1, or ICAM‐1, is a membrane‐bound glycoprotein that plays a key role in various immune system processes such as lymphocyte activation and leukocyte migration during an immune response.^47^, ^48^, ^49^ Thus, the upregulation of ICAM‐1 (as observed in Figure 3A,B) is a signature event that occurs during inflammation, vascular inflammation and throughout the inflammatory response. Our findings illustrate significant upregulation of ICAM‐1 in the presence of C1q, indicating a potential relationship between the complement system and the TNF‐α pathway.^49^ Although C1q has not directly been shown to be associated with this relationship, other links between complement and ICAM‐1 have been observed. For example, C5b and the membrane attack complex (MAC) have both been shown to enhance ICAM‐1 expression in endothelial cells.^50^, ^51^ Our results confirm these findings and show additional relationships between complement and ICAM‐1 expression.

### 4.3 Tissue factor expression while blocking gC1qR

The expression of tissue factor in both AFs and SMCs was assessed to determine if the previously observed elevated expression in TF by C1q was through the binding of C1q to gC1qR. Our findings illustrate no changes in TF expression when the binding of C1q to its receptor, gC1qR, is blocked in both studied cell types (Figure 4A,B). This indicates that that gC1qR is necessary in C1q‐mediated TF expression. It is well‐known that gC1qR serves as a receptor for the globular head of C1q^18^, ^19^, ^52^ and that the epitope of gC1qR within amino acids 76 to 93 (60.11 antibody binding domain) is the site at which C1q associates with gC1qR.^53^, ^54^ We assessed the changes in TF expression while blocking with an antibody toward 60.11 and observed no changes in TF expression in either cell type. Thus, our data supports the role of the C1q‐gC1qR axis on tissue factor expression within sub‐endothelial cells. As there was no direct previous work that we can compare with, our findings indicate a new link between inflammatory and thrombotic changes. It is important to remember that C1q‐gC1qR association has been linked with intrinsic coagulation activity^55^ and thus, we now illustrate that there is a relationship between extrinsic coagulation and C1q‐gC1qR activity.

### 4.4 Secreted tissue factor

We also investigated the potential for C1q‐gC1qR activity to increase the secretion of tissue factor from AFs and SMCs and whether or not pharmacologically blocking this association alters tissue factor secretion (Figure 5). There were no changes in the secretion of tissue factor as a function of exposure to C1q or the blocking antibody, illustrating that any potential changes in extrinsic coagulation due to C1q exposure, would be induced by changes in membrane bound tissue factor. To the best of our knowledge, no previous work quantified the secretion of tissue factor as a function in C1q exposure, under any relevant comparison conditions.

## 5 CONCLUSION AND STUDY LIMITATIONS

**Figure 6:**
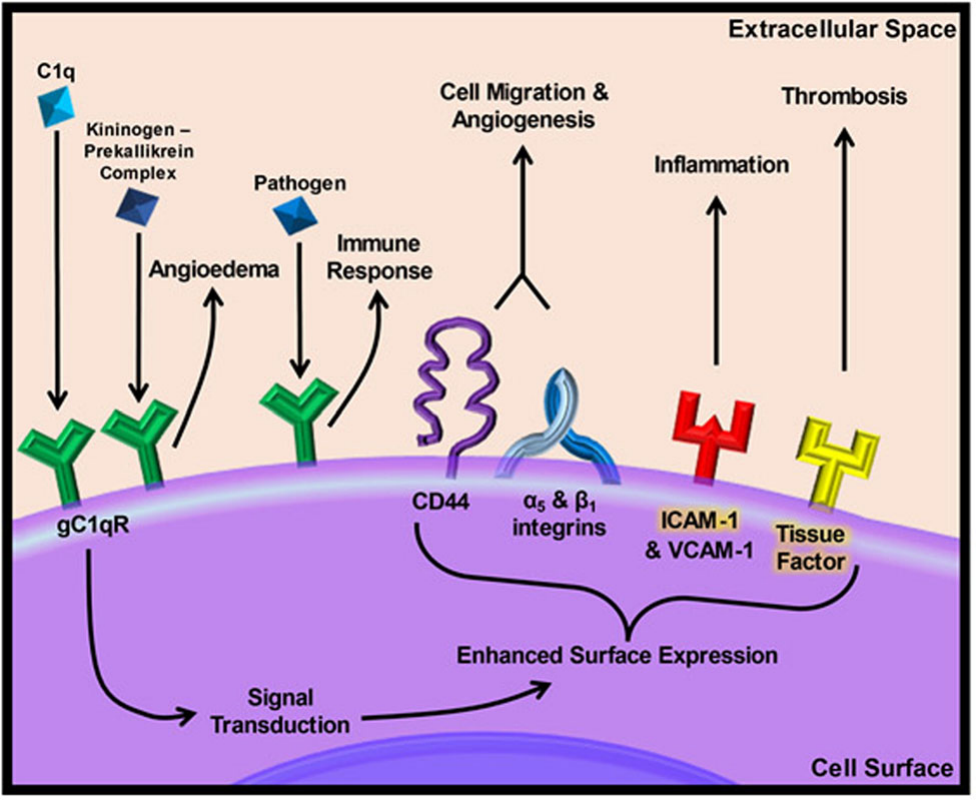
Model of gC1qR activation and the physiological and pathological responses that can be observed upon gC1qR activation. Traditionally, altered gC1qR activity has been associated with angioedema (via high molecular weight kininogen and prekallikrein activity) and heightened innate immune responses (e.g., complement activation). However, more recent work has shown that gC1qR activity is associated with migration, angiogenesis, cancer, intrinsic coagulation (through factor XII activation) and general inflammatory responses. The work described in this report extends the known role of gC1qR by showing that C1q association with gC1qR can lead to enhanced tissue factor expression, which can potentially lead to activation of the extrinsic arm of the coagulation cascade and pathological thrombosis. Receptors names that we investigated are highlighted.

It has become apparent that C1q and the complement system, in general, are strongly linked to inflammation, thrombosis, and cardiovascular complications. However, a complete understanding of this interrelationship has yet to be elucidated. Thus, we aimed to investigate whether or not there is a link between C1q association with gC1qR and tissue factor expression in subendothelial cells. Combining our data, we illustrate that a short‐term exposure to C1q induces a rapid and significant change in the expression of tissue factor, which is the rate‐limiting step for extrinsic coagulation initiation. Our data provides means to further investigate the intracellular mechanism behind the expression of tissue factor as a result of C1q/gC1qR interactions as well as the downstream effects on the extrinsic coagulation cascade. However, it must be noted that our study is limited by the model system that we have employed, the short duration exposure to C1q and assessment of receptor expression. Even with these limitations it is important to note that we still observed, for the first time, the convergence of innate inflammatory signals with extrinsic coagulation, via gC1qR activity. Determining the relevance of this link in vivo is important for future studies. It is also important to note that if gC1qR can serve as a convergent receptor for inflammation and coagulation, then the role of gC1qR in disease processes, the ability to therapeutically target gC1qR, and downstream activation of the common coagulation cascade must be investigated. It is possible that gC1qR can serve as a new therapeutic target to minimize diseases characterized by vascular inflammatory and thrombotic processes. A summary figure illustrating the known and newly identified roles of gC1qR and the potential outcomes of antagonizing these pathways has been prepared (Figure 6).

## AUTHOR CONTRIBUTIONS

Christopher T. Freda performed the research. Christopher T. Freda, Wei Yin, Berhane Ghebrehiwet and David A. Rubenstein designed the research study. Christopher T. Freda and David A. Rubenstein analyzed the data. Wei Yin, Berhane Ghebrehiwet and David A. Rubenstein secured research funding for this work described. All authors wrote and approved the final manuscript.

## CONFLICTS OF INTEREST

The authors (B. G.) receive royalties from the sale of monoclonal antibodies against gC1qR clone 60.11. The authors (B. G.) hold a patent for the development of these antibodies for therapy against cancer and angioedema, respectively (US patent 8,883,153‐B2, “Methods for Prevention and Treatment of Angioedema”).

## Data Availability

The data that support the findings of this study are available from the corresponding author upon reasonable request.

## References

[1] Hajar R . Coronary heart disease: from mummies to 21(st) century. Heart Views. 2017;18(2):68‐74. 10.4103/heartviews.heartviews_57_17 28706602 PMC5501035

[2] Boudoulas KD , Triposkiadis F , Gumina R , Addison D , Iliescu C , Boudoulas H . Cardiovascular disease, cancer, and multimorbidity interactions: clinical implications. Cardiology. 2022;147(2):196‐206. 10.1159/000521680 34986484

[3] Lusis AJ . Atherosclerosis. Nature. 2000;407(6801):233‐241. 10.1038/35025203 11001066 PMC2826222

[4] Galkina E , Ley K . Immune and inflammatory mechanisms of atherosclerosis (*). Annu Rev Immunol. 2009;27:165‐197. 10.1146/annurev.immunol.021908.132620 19302038 PMC2734407

[5] Raghunathan S , Rayes J , Sen Gupta A . Platelet‐inspired nanomedicine in hemostasis thrombosis and thromboinflammation. J Thromb Haemostasis. 2022;20(7):1535‐1549. 10.1111/jth.15734 35435322 PMC9323419

[6] Palta S , Saroa R , Palta A . Overview of the coagulation system. Indian J Anaesth. 2014;58(5):515‐523. 10.4103/0019-5049.144643 25535411 PMC4260295

[7] Mackman N . The role of tissue factor and factor VIIa in hemostasis. Anesth Analg. 2009;108(5):1447‐1452. 10.1213/ane.0b013e31819bceb1 19372318 PMC2838713

[8] Lakshmanan HHS , Estonilo A , Reitsma SE , et al. Revised model of the tissue factor pathway of thrombin generation: role of the feedback activation of FXI. J Thromb Haemostasis. 2022;20(6):1350‐1363. 10.1111/jth.15716 35352494 PMC9590754

[9] Branchford BR , Carpenter SL . The role of inflammation in venous thromboembolism. Front Pediatr. 2018;6:142. 10.3389/fped.2018.00142 29876337 PMC5974100

[10] Speer T , Dimmeler S , Schunk SJ , Fliser D , Ridker PM . Targeting innate immunity‐driven inflammation in CKD and cardiovascular disease. Nat Rev Nephrol. 2022;18(12):762‐778. 10.1038/s41581-022-00621-9 36064794

[11] Nesargikar PN , Spiller B , Chavez R . The complement system: history, pathways, cascade and inhibitors. Eur J Microbiol Immunol. 2012;2(2):103‐111. 10.1556/EuJMI.2.2012.2.2 PMC3956958 24672678

[12] Dunkelberger JR , Song W‐C . Complement and its role in innate and adaptive immune responses. Cell Res. 2010;20(1):34‐50. 10.1038/cr.2009.139 20010915

[13] Ghebrehiwet B . cC1q‐R (calreticulin) and gC1q‐R/p33: ubiquitously expressed multi‐ligand binding cellular proteins involved in inflammation and infection. Mol Immunol. 2004;41(2):173‐183. 10.1016/j.molimm.2004.03.014 15159063

[14] Chowdhury AR , Ghosh I , Datta K . Excessive reactive oxygen species induces apoptosis in fibroblasts: role of mitochondrially accumulated hyaluronic acid binding protein 1 (HABP1/p32/gC1qR. Exp Cell Res. 2008;314(3):651‐667. 10.1016/j.yexcr.2007.10.033 18166172

[15] Hosszu KK , Santiago‐Schwarz F , Peerschke EIB , Ghebrehiwet B . Evidence that a C1q/C1qR system regulates monocyte‐derived dendritic cell differentiation at the interface of innate and acquired immunity. Innate Immun. 2010;16(2):115‐127. 10.1177/1753425909339815 19710097 PMC2846191

[16] Peerschke EIB , Ghebrehiwet B . The contribution of gC1qR/p33 in infection and inflammation. Immunobiology. 2007;212(4‐5):333‐342. 10.1016/j.imbio.2006.11.011 17544818 PMC2001281

[17] Kishore U , Reid KBM . C1q: structure, function, and receptors. Immunopharmacology. 2000;49(1):159‐170. 10.1016/S0162-3109(00)80301-X 10904115

[18] Pednekar L , Pathan AA , Paudyal B , et al. Analysis of the interaction between globular head modules of human C1q and its candidate receptor gC1qR. Front Immunol. 2016;7:567. 10.3389/fimmu.2016.00567 28018340 PMC5153404

[19] Ghebrehiwet B , Jesty J , Vinayagasundaram R , et al. Targeting gC1qR domains for therapy against infection and inflammation. Adv Exp Med Biol. 2013;735:97‐110. 10.1007/978-1-4614-4118-2_6 23402021

[20] Freda CT , Yin W , Ghebrehiwet B , Rubenstein DA . SARS‐CoV‐2 proteins regulate inflammatory, thrombotic and diabetic responses in human arterial fibroblasts. Clin Immunol. 2021;227:108733. 10.1016/j.clim.2021.108733 33895357 PMC8061629

[21] Freda CT , Yin W , Ghebrehiwet B , Rubenstein DA . SARS‐CoV‐2 structural proteins exposure alter thrombotic and inflammatory responses in human endothelial cells. Cell Mol Bioeng. 2021;15:43‐53. 10.1007/s12195-021-00696-7 34484458 PMC8407404

[22] Kostner KM , Fahti RB , Case C , Hobson P , Tate J , Marwick TH . Inflammation, complement activation and endothelial function in stable and unstable coronary artery disease. Clin Chim Acta. 2006;365(1‐2):129‐134. 10.1016/j.cca.2005.08.028 16236275

[23] Yasojima K , Schwab C , McGeer EG , McGeer PL . Complement components, but not complement inhibitors, are upregulated in atherosclerotic plaques. Arterioscler Thromb Vasc Biol. 2001;21(7):1214‐1219. 10.1161/hq0701.092160 11451754

[24] Peerschke E , MINTA J , ZHOU S , et al. Expression of gC1q‐R/p33 and its major ligands in human atherosclerotic lesions. Mol Immunol. 2004;41(8):759‐766. 10.1016/j.molimm.2004.04.020 15234555

[25] Oikonomopoulou K , Ricklin D , Ward PA , Lambris JD . Interactions between coagulation and complement‐‐their role in inflammation. Semin Immunopathol. 2012;34(1):151‐165. 10.1007/s00281-011-0280-x 21811895 PMC3372068

[26] Ruf W . Links between complement activation and thrombosis. Blood. 2019;134‐SCI‐40. 10.1182/blood-2019-121113

[27] Yin W , Ghebrehiwet B , Weksler B , Peerschke EI . Classical pathway complement activation on human endothelial cells. Mol Immunol. 2007;44(9):2228‐2234. 10.1016/j.molimm.2006.11.012 17173972 PMC1865514

[28] Aukrust P , Gullestad L , Lappegård KT , et al. Complement activation in patients with congestive heart failure. Circulation. 2001;104(13):1494‐1500. 10.1161/hc3801.096353 11571242

[29] Sumida T , Naito AT , Nomura S , et al. Complement C1q‐induced activation of β‐catenin signalling causes hypertensive arterial remodelling. Nat Commun. 2015;6:6241. 10.1038/ncomms7241 25716000 PMC4351572

[30] Rubenstein DA , Hom S , Ghebrehiwet B , Yin W . Tobacco and e‐cigarette products initiate Kupffer cell inflammatory responses. Mol Immunol. 2015;67(2 pt B):652‐660. 10.1016/j.molimm.2015.05.020 26072673

[31] Yin W , Ngwe EC , Ghebrehiwet B , Rubenstein DA . The combined effect of sidestream smoke and dynamic shear stress on endothelial cell inflammatory responses. Thromb Res. 2015;135(2):362‐367. 10.1016/j.thromres.2014.11.018 25467082

[32] Naito AT , Sumida T , Nomura S , et al. Complement C1q activates canonical Wnt signaling and promotes aging‐related phenotypes. Cell. 2012;149(6):1298‐1313. 10.1016/j.cell.2012.03.047 22682250 PMC3529917

[33] Hong Q , Sze CI , Lin SR , et al. Complement C1q activates tumor suppressor WWOX to induce apoptosis in prostate cancer cells. PLoS One. 2009;4(6):e5755. 10.1371/journal.pone.0005755 19484134 PMC2685983

[34] Shingu M , Yoshioka K , Nobunaga M , Motomatu T . C1q binding to human vascular smooth muscle cells mediates immune complex deposition and superoxide generation. Inflammation. 1989;13(5):561‐569. 10.1007/bf00916762 2553603

[35] Bordin S , Tan X . C1q arrests the cell cycle progression of fibroblasts in G1 phase: role of the cAMP/PKA‐I pathway. Cellular Signalling. 2001;13(2):119‐123. 10.1016/S0898-6568(00)00139-X 11257456

[36] Nauta AJ , Trouw LA , Daha MR , et al. Direct binding of C1q to apoptotic cells and cell blebs induces complement activation. Eur J Immunol. 2002;32(6):1726‐1736. 10.1002/1521-4141(200206)32:6<1726::aid-immu1726>3.0.co;2-r 12115656

[37] Ling GS , Crawford G , Buang N , et al. C1q restrains autoimmunity and viral infection by regulating CD8(+) T cell metabolism. Science. 2018;360(6388):558‐563. 10.1126/science.aao4555 29724957 PMC6545171

[38] Tan X , Wong ST , Ghebrehiwet B , Storm DR , Bordin S . Complement C1q inhibits cellular spreading and stimulates adenylyl cyclase activity of fibroblasts. Clin Immunol Immunopathol. 1998;87(2):193‐204. 10.1006/clin.1997.4485 9614935

[39] Esmon CT . The impact of the inflammatory response on coagulation. Thromb Res. 2004;114(5):321‐327. 10.1016/j.thromres.2004.06.028 15507261

[40] Markiewski MM , Nilsson B , Nilsson Ekdahl K , Mollnes TE , Lambris JD . Complement and coagulation: strangers or partners in crime. Trends Immunol. 2007;28(4):184‐192. 10.1016/j.it.2007.02.006 17336159

[41] Markiewski MM , Lambris JD . The role of complement in inflammatory diseases from behind the scenes into the spotlight. Am J Pathol. 2007;171(3):715‐727. 10.2353/ajpath.2007.070166 17640961 PMC1959484

[42] Huber‐Lang M , Sarma JV , Zetoune FS , et al. Generation of C5a in the absence of C3: a new complement activation pathway. Nature Med. 2006;12(6):682‐687. 10.1038/nm1419 16715088

[43] Amara U , Flierl MA , Rittirsch D , et al. Molecular intercommunication between the complement and coagulation systems. J Immunol. 2010;185(9):5628‐5636. 10.4049/jimmunol.0903678 20870944 PMC3123139

[44] Ghebrehiwet B , Silverberg M , Kaplan AP . Activation of the classical pathway of complement by Hageman factor fragment. J Exp Med. 1981;153(3):665‐676. 10.1084/jem.153.3.665 7252410 PMC2186101

[45] Polley MJ , Nachman R . The human complement system in thrombin‐mediated platelet function. J Exp Med. 1978;147(6):1713‐1726. 10.1084/jem.147.6.1713 681879 PMC2184322

[46] Peerschke EI , Reid KB , Ghebrehiwet B . Platelet activation by C1q results in the induction of alpha IIb/beta 3 integrins (GPIIb‐IIIa) and the expression of P‐selectin and procoagulant activity. J Exp Med. 1993;178(2):579‐587. 10.1084/jem.178.2.579 7688027 PMC2191135

[47] Ramos TN , Bullard DC , Barnum SR . ICAM‐1: isoforms and phenotypes. J Immunol. 2014;192:4469‐4474. 10.4049/jimmunol.1400135 24795464 PMC4015451

[48] Wiesolek HL , Bui TM , Lee JJ , et al. Intercellular adhesion molecule 1 functions as an efferocytosis receptor in inflammatory macrophages. Am J Pathol. 2020;190(4):874‐885. 10.1016/j.ajpath.2019.12.006 32035057 PMC7180595

[49] Frank PG , Lisanti MP . ICAM‐1: role in inflammation and in the regulation of vascular permeability. Am J Physiol Heart Circ Physiol. 2008;295(3):H926‐H927. 10.1152/ajpheart.00779.2008 18689494 PMC2544488

[50] Skeie JM , Fingert JH , Russell SR , Stone EM , Mullins RF . Complement component C5a activates ICAM‐1 expression on human choroidal endothelial cells. Invest Ophthalmol Visual Sci. 2010;51(10):5336‐5342. 10.1167/iovs.10-5322 20484595 PMC3066598

[51] Kilgore KS , Shen JP , Miller BF , Ward PA , Warren JS . Enhancement by the complement membrane attack complex of tumor necrosis factor‐alpha‐induced endothelial cell expression of E‐selectin and ICAM‐1. J Immunol. 155(3), 1995:1434‐1441. 7543521

[52] Jiang J , Zhang Y , Krainer AR , Xu RM Crystal structure of human p32, a doughnut‐shaped acidic mitochondrial matrix protein. *Proceedings of the National Academy of Sciences of the United States of America*. 1999;96(7):3572‐3577. 10.1073/pnas.96.7.3572 PMC22335 10097078

[53] Ghebrehiwet B , Feng X , Kumar R , Peerschke EIB . Complement component C1q induces endothelial cell adhesion and spreading through a docking/signaling partnership of C1q receptors and integrins. Int Immunopharmacol. 2003;3(3):299‐310. 10.1016/s1567-5769(02)00270-9 12639807

[54] Peerschke EIB , Bayer AS , Ghebrehiwet B , Xiong YQ . gC1qR/p33 blockade reduces *Staphylococcus aureus* colonization of target tissues in an animal model of infective endocarditis. Infect Immun. 2006;74(8):4418‐4423. 10.1128/IAI.01794-05 16861627 PMC1539591

[55] Peerschke EIB , Jesty J , Reid KBM , Ghebrehiwet B . The soluble recombinant form of a binding protein/receptor for the globular domain of C1q (gC1qR) enhances blood coagulation. Blood Coagul Fibrinolysis. 1998;9(1):29‐38. 10.1097/00001721-199801000-00004 9607116

